# Unchecked Immunity to Unleashed Anarchy: A Case of Hemophagocytic Lymphohistiocytosis Following Combination Immune Checkpoint Inhibitor Therapy

**DOI:** 10.7759/cureus.90533

**Published:** 2025-08-19

**Authors:** Sunil R Dommaraju, Dimitrios Stefanoudakis, Gavin Hui, Alexandra Drakaki

**Affiliations:** 1 Department of Medicine, Division of General Internal Medicine, University of California Los Angeles (UCLA) Ronald Reagan Medical Center, Los Angeles, USA; 2 Department of Medicine, Division of Hematology-Oncology, University of California Los Angeles (UCLA) Ronald Reagan Medical Center, Los Angeles, USA

**Keywords:** clear-cell renal carcinoma, hemophagocytic lymphohistiocytosis (hlh), h-score, immune-checkpoint inhibitors, immune-related adverse effects

## Abstract

Immune checkpoint inhibitors (ICIs) can cause rare but fatal immune-related adverse events such as hemophagocytic lymphohistiocytosis (HLH). We describe a 42-year-old female with metastatic clear cell renal cell carcinoma who developed HLH following treatment with first-line ipilimumab and nivolumab. Initial symptoms mimicked sepsis and recurrent *Clostridioides difficile* infection, delaying diagnosis. Eventually, laboratory findings met HLH-2004 criteria, and initiation of high-dose corticosteroids led to improvement. This case illustrates the diagnostic complexity of HLH in ICI-treated patients and the importance of early recognition. As ICI use increases, awareness of HLH is critical. Standardized treatment protocols and prospective studies are needed to guide management of this rare but severe complication.

## Introduction

Immune checkpoint inhibitors (ICIs) have dramatically altered the landscape of cancer treatment for multiple tumor types, where they have significantly improved survival outcomes [1]. ICIs work by blocking immune checkpoint proteins that regulate T-cell activity, like cytotoxic T-lymphocyte antigen 4 (CTLA-4) and programmed death-ligand 1 (PD-L1) [2]. CTLA-4 primarily acts during the initial priming of T-cells in lymph nodes, while PD-L1 primarily acts in peripheral tissues [2]. Targeting these proteins thereby reactivates T-cell responses against tumor cells. However, the use of ICIs is frequently complicated by on-target off-tumor immune-related adverse events (irAEs), which are the result of the overstimulation of the immune system and can affect nearly any organ system [3]. While most irAEs are manageable, severe hyperinflammatory syndromes, including hemophagocytic lymphohistiocytosis (HLH), have emerged as life-threatening complications. HLH, a disorder of excessive immune activation, is characterized by cytokine storm, tissue damage, and multi-organ failure, and its incidence in patients receiving ICIs is increasingly recognized [4-5]. Given the overlap between HLH and other causes of fever and organ dysfunction in ICI-treated patients, early recognition and differentiation from infections, disease progression, or other immune-mediated conditions are crucial for their survival. Here, we describe the clinical trajectory and medical enigma of a patient with clear cell renal cell carcinoma treated with a combination of a programmed cell death protein 1 (PD-1)/CTLA4 inhibitor who had a sepsis-like presentation but was eventually diagnosed with HLH secondary to the ICI.

## Case presentation

A 42-year-old female with a five-month history of clear cell renal cell carcinoma initially managed with left radical nephrectomy, but with recurrence ultimately at the nephrectomy bed along with regional and distant metastases to the liver, presented to the emergency department with fevers and generalized body aches a week after her second cycle of the first-line therapy with nivolumab and ipilimumab. With a positive urinalysis for leukocyte esterase, she was given IV broad-spectrum antibiotics. She was discharged home on an empiric course of oral cefdinir 300 mg twice a day to complete the treatment for what was suspected to be a urinary tract infection. Despite supportive measures, her clinical presentation then evolved to one week of high fevers and chills, foul-smelling and cloudy urine (with continued positive leukocyte esterase on urinalysis), profuse diaphoresis, and pruritus. At that point, she re-presented to the emergency department.

On initial evaluation, vital signs were a temperature of 103.1°F, blood pressure of 122/85 mmHg, and tachycardia at 141 bpm, as seen in Table 1. The exam revealed erythematous patches and dry mucous membranes, but the patient had no focal neurological deficits, abdominal tenderness or distention, or irregular sounds on pulmonary or cardiac auscultation.

**Table 1:** Vital signs on day 1 and day 3 of hospitalization

Hospital day	Temperature (^o^F)	Heart rate (bpm)	Blood pressure (mmHg)	Respiratory rate (breaths/min)
1	103.1	141	122/85	22
3	103.1	125	101/67	21

Labs showed normal white blood cell count, anemia, thrombocytopenia, lactic acidosis, and new-onset transaminitis (Table 2).

**Table 2:** Lab values on day of admission

Test	Baseline values (9 days prior)	Results (day 1)	Reference range
Hemoglobin	10.5	9.1	11.6-15.2 g/dL
White blood cell count	6.55	6.46	4.16-9.95 k/uL
Platelets	448	191	143-398 k/uL
Mean corpuscular volume	77.2	73.9	79.3-98.6 fL
Creatinine	0.93	1.05	0.60-1.30 mg/dL
Total CO2 (bicarbonate)	24	15	20-30 mmol/L
Lactate	Not available	37	5-18 mg/dL
Anion gap	15	20	8-19 mmol/L
Aspartate aminotransferase	56	242	13-62 U/L
Alanine transaminase	88	308	8-70 U/L
Alkaline phosphatase	126	192	37-113 U/L
Total bilirubin	<0.2	0.4	0.1-1.2 mg/dL

A CT of the abdomen and pelvis revealed mild bowel wall thickening, progression in baseline hepatic metastases, and new splenomegaly. The patient was admitted with severe sepsis secondary to a presumed urinary tract infection versus infectious colitis.

Fluids and empiric broad-spectrum antibiotics (vancomycin and meropenem) were initiated but discontinued after 48 hours, given an entirely negative infectious work-up. New watery diarrhea (> 3 episodes daily) and abdominal bloating on hospital day 2 prompted stool studies, including a bacterial enteric pathogen panel, an ova and parasite panel, and *Clostridium difficile* (*C. Diff*) toxin antigen, especially given the patient had a hospitalization for *C. Diff* colitis two months prior, which was successfully treated with oral fidaxomicin. Stool studies during this admission confirmed recurrent *C. Diff* infection, so oral fidaxomicin 200 mg twice a day was restarted.

Persistent fever (max 103.1°F) and tachycardia (110-120 bpm) on hospital day 3 despite treatment for the infectious colitis led to evaluation for hyperimmune system activation. Labs showed elevated ferritin from baseline, high soluble interleukin-2 receptor level, low natural killer cell activity, mildly elevated triglycerides, and normal fibrinogen (Table 3). Meeting five of eight diagnostic criteria, she was diagnosed with HLH secondary to ICI therapy.

**Table 3:** Features of HLH and patient values H-score: a scoring system used to assess the probability of HLH, CD: cluster of differentiation, AST: aspartate aminotransferase, ALT: alanine transaminase, ALP: alkaline phosphatase, HLH: hemophagocytic lymphohistiocytosis

Feature	Cutoff value	Included in H-score	Patient values on day 3	Meets criteria
Fever	≥38.5°C	Yes	39.5°C	Yes
Splenomegaly	≥2 cm below the costal margin	Yes	Yes	Yes
Cytopenia	≥2 of the cell lines below: hemoglobin <9 g/dL, platelets <100 × 10^3^/uL, and neutrophils <1 × 10^3^/ uL	Yes	Hemoglobin: 7.7 g/dL, platelets: 172 × 10^3^/uL, neutrophils: 3.64 × 10^3^/uL	No
Hypofibrinogenemia or hypertriglyceridemia	Fibrinogen level of ≤150 mg/dL or triglyceride level of ≥3.0 mmol/liter	Yes	Fibrinogen: 353 mg/dL, triglycerides: 224 mg/dL	No
Hyperferritinemia	≥500 ng/mL	Yes	3,659 ng/mL	Yes
Hemophagocytosis	Bone marrow, other tissues	Yes	Not performed	N/A
Elevated soluble CD25	≥2400 U/mL	No	7,005 U/mL	Yes
Natural killer cell activity	Reduced or absent	No	Reduced	Yes
Other features				
Hepatomegaly	N/A	Yes	Yes	Yes
Elevated aminotransferases	N/A	Yes	AST: 248 U/L, ALT: 255 U/L, ALP: 170 U/L	Yes
Known underlying immunosuppression	N/A	Yes	Yes	Yes

The patient was initiated on IV methylprednisolone at a dose of 0.8 mg per kilogram on hospital day 4 with complete resolution of the fevers. With improvement in the ferritin, C-reactive protein, and lactate dehydrogenase, a confirmatory bone marrow biopsy was deferred. She was discharged on hospital day 6 with 80 mg of prednisone daily; however, she developed recurrent fevers, transaminitis, and worsening of the inflammatory markers, so her prednisone dose was doubled to 150 mg daily at an outpatient visit one week after discharge. Over the next two months, she showed clinical and laboratory improvement, so her steroids were tapered and subsequently discontinued. Given the direct association of her presentation with ICI therapy and her radiographic disease progression, her cancer-directed therapy was changed to the combination of lenvatinib and everolimus, a vascular epidermal growth factor inhibitor and a mammalian target of rapamycin inhibitor, respectively, that are approved in the second-line setting.

## Discussion

**Figure 1:**
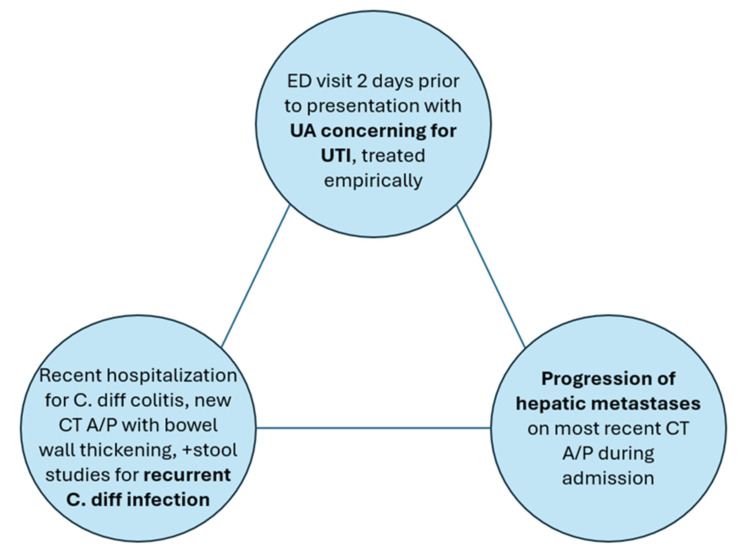
Clinical confounders ED: emergency department, UA: urinalysis, UTI: urinary tract infection, CT A/P: computed tomography of the abdomen and pelvis, *C. Diff*: *Clostridioides difficile*

We presented the case of a 42-year-old female with widespread metastatic clear cell renal carcinoma who presented with clinical signs of sepsis but, after failure to improve with empiric antibiotics, was diagnosed with HLH secondary to ICI therapy. Features that delayed diagnosis in this case include the history of recent hospitalization for infectious *C. Diff* colitis, the CT of abdomen and pelvis findings suggestive of colitis, and the positive stool test for *C. Diff*, which made infectious *C. Diff* colitis a clear distracting diagnosis on the differential. Similarly, the patient had foul-smelling urine, with a recent emergency department visit anchoring the diagnosis in a possible urinary tract infection. Finally, the progression of the patient’s liver metastases on CT imaging was a confounder for the finding of transaminitis. These confounders, summarized in Figure 1, rightfully led the clinical team to manage the patient for sepsis initially; however, the recent ICI therapy was a highly relevant clinical factor that allowed swift evaluation for HLH.

HLH is a rare and life-threatening disease. It is the result of immune system activation, causing a hyperinflammatory state and multi-organ failure. It is diagnosed based on a combination of clinical and laboratory criteria, including fever, splenomegaly, cytopenias, hyperferritinemia, and elevated soluble cluster of differentiation 25 levels, with a definitive diagnosis requiring the presence of at least five of eight criteria as seen in Table 3 [6-7]. The most distinctive feature is a ferritin level above 500 ng/mL; however, it is not uncommon to have even more pronounced elevations. Ferritin levels above 1000 ng/mL firmly indicate HLH disease in the right clinical context. The disease can be classified into various subtypes and should be distinguished from disease mimics [8]. Notably, primary HLH is associated with genetic mutations. On the other hand, secondary HLH could be triggered by treatment such as ICIs, infection, or diseases such as Epstein-Barr virus, bacterial and fungal infections, autoimmune or autoinflammatory conditions, and cancer, respectively [9].

The exact pathophysiology of HLH differs based on the inciting trigger. For primary HLH, defective cytotoxicity of cytotoxic T-lymphocytes and natural killer cells results in a massive release of cytokines. In ICI therapy, some articles report that HLH incidence is more frequent with the use of CTLA-4 inhibitors in comparison to PD(L)-1 inhibitors for mechanisms that remain unknown [2]. One suggested theory is that CTLA-4 blockade occurs earlier in the immune response than do anti-PD-1 agents, allowing for more widespread immune activation that ultimately leads to the immune hyperactivity of HLH [10].

The diagnosis of HLH in the context of ICI therapy presents significant clinical challenges due to its nonspecific presentation. Symptoms often overlap with common inflammatory responses, infections, or even disease progression, leading to delays in diagnosis and treatment [4]. Patients may present with fever unresponsive to antibiotics, hepatomegaly, splenomegaly, rash, enlarged lymph nodes, jaundice, pale skin, weakness, anemia, thrombocytopenia, dizziness, and headaches. At the same time, some of the serious signs of HLH are dyspnea, seizures, retinal hemorrhages, loss of consciousness, and even coma [11]. The most common symptom reported is fever, which affects more than 90% of patients and usually presents as the initial clinical indicator [5]. Among 22 reported HLH cases secondary to ICIs, fever, cytopenias, and elevated ferritin were the most common findings [5]. The time between starting immunotherapy and symptom onset varies widely from several days to multiple years. HLH incidence in a clinical trial of 6000 patients treated with ICIs was only 0.4%, and there was a 14% mortality rate across all hematologic irAEs [12]. Still, any patient on ICIs with non-infectious, recurrent fevers, cytopenias, and/or ferritin elevation should have HLH on their differential diagnosis.

The timely initiation of high-dose corticosteroids remains the cornerstone of treatment for HLH secondary to ICIs. Steroids can often be used alone, especially given comparable mortality in cases where both steroids and immunomodulators were used [4]. Methylprednisolone is typically used at doses of 1-2 mg/kg/day or equivalent pulse dosing, and clinical improvement is often seen within several days if HLH is steroid-responsive [13-14]. In steroid-refractory cases or those with severe or rapidly progressive disease, second-line therapies such as etoposide, IV immunoglobulin, anakinra (interleukin-1 receptor antagonist), and tocilizumab (interleukin-6 inhibitor) have been used. Once clinical and laboratory improvement is observed, steroids should be gradually tapered over several weeks to months, guided by ferritin trends and inflammatory markers, to prevent relapse as seen in our case. The HLH-94 protocol, which includes etoposide and dexamethasone, may be considered in life-threatening cases. However, the immunosuppressive burden must be weighed against the underlying oncologic diagnosis, bone marrow reserve, performance status, and overall prognosis. Given the severity of HLH and the expanding use of ICIs, there is a need for standardized treatment algorithms and prospective studies to define optimal immunosuppressive regimens and tapering strategies better.

There are four reports of patients being rechallenged with an ICI after HLH resolved, and only one had recurrent HLH that was less severe; in the recurrent case, the recurrence happened 10 days after their initial nivolumab dose and quickly improved with IV steroids [4]. Ultimately, ICI re-treatment should only be reserved for patients with no other treatment options and must involve meticulous attention to monitoring.

## Conclusions

Immunotherapy has revolutionized cancer treatment, but early recognition and management of irAEs, such as HLH, remain challenging. Given that data specific to ICI-induced HLH are limited to case reports and small series, the morbidity and mortality associated with this condition are not insignificant. Given that the use of ICIs is expanding, there is a need for prospective studies on how best to diagnose and treat this rare irAE in a timely and effective manner. This case report contributes to the body of literature by providing important clinical information in terms of the presentation, time course, and treatment strategies for HLH secondary to ICIs. There is a strong need for enriched and clear algorithms related to steroid tapering with or without immunosuppressive regimens. Hopefully, as we all share our clinical experience and practices with the medical community, our knowledge will broaden, and patient care will improve.
